# Medical Students in Philadelphia

**Published:** 1860-01

**Authors:** 


Medical Students in Philadelphia.—The number of medical students in this city, this winter, is considerably upwards of twelve hundred. We have no definite information of the size of the classes in other parts of the Union.

